# Robust Action Recognition Using Multi-Scale Spatial-Temporal Concatenations of Local Features as Natural Action Structures

**DOI:** 10.1371/journal.pone.0046686

**Published:** 2012-10-04

**Authors:** Xiaoyuan Zhu, Meng Li, Xiaojian Li, Zhiyong Yang, Joe Z. Tsien

**Affiliations:** 1 Brain and Behavior Discovery Institute, Medical College of Georgia, Georgia Regents University, Augusta, Georgia, United States of America; 2 Department of Neurology, Medical College of Georgia, Georgia Regents University, Augusta, Georgia, United States of America; 3 Department of Ophthalmology, Medical College of Georgia, Georgia Regents University, Augusta, Georgia, United States of America; Max Planck Institute for Human Cognitive and Brain Sciences, Germany

## Abstract

Human and many other animals can detect, recognize, and classify natural actions in a very short time. How this is achieved by the visual system and how to make machines understand natural actions have been the focus of neurobiological studies and computational modeling in the last several decades. A key issue is what spatial-temporal features should be encoded and what the characteristics of their occurrences are in natural actions. Current global encoding schemes depend heavily on segmenting while local encoding schemes lack descriptive power. Here, we propose natural action structures, i.e., multi-size, multi-scale, spatial-temporal concatenations of local features, as the basic features for representing natural actions. In this concept, any action is a spatial-temporal concatenation of a set of natural action structures, which convey a full range of information about natural actions. We took several steps to extract these structures. First, we sampled a large number of sequences of patches at multiple spatial-temporal scales. Second, we performed independent component analysis on the patch sequences and classified the independent components into clusters. Finally, we compiled a large set of natural action structures, with each corresponding to a unique combination of the clusters at the selected spatial-temporal scales. To classify human actions, we used a set of informative natural action structures as inputs to two widely used models. We found that the natural action structures obtained here achieved a significantly better recognition performance than low-level features and that the performance was better than or comparable to the best current models. We also found that the classification performance with natural action structures as features was slightly affected by changes of scale and artificially added noise. We concluded that the natural action structures proposed here can be used as the basic encoding units of actions and may hold the key to natural action understanding.

## Introduction

Human and many other animals can easily detect, recognize, and classify a range of actions very quickly. How this is accomplished by the visual system has been intensively studied over the past several decades [1]–[3]. Extensive studies suggest that both the ventral and dorsal streams of visual cortex are involved in the processing of human or animal actions [4], [5]. Along the hierarchical visual pathways, neurons at early levels have small receptive fields and encode simple features such as orientation and direction of motion. Neurons at higher levels have large receptive fields and encode complex features such as complex shapes and motion trajectories. These neurobiological studies motivated, to varying degrees, a number of computational models of human action understanding [5]–[10]. Because of its many applications in surveillance, human-machine interaction, and robotics, action recognition and understanding resurges as an active research area of computer vision.

A key issue of action recognition is what spatial-temporal features should be used. There are two broad feature representations of actions, global representations and local representations. In global representations [11], [12], actions are segmented from background first and space-time volumes extracted from the segmented videos are used for recognition. Global representations can be highly informative, but they depend heavily on segmentation and tracking, and are sensitive to changes of viewpoints and occlusions. In local representations [13]–[16], a set of local, multi-scale features compiled at the selected points of interest, including cuboids descriptors [17], histograms of gradient orientation or optic flow [18], extended speeded up robust features [19], are used for action recognition. Local representations are not sensitive to changes of viewpoints and occlusions, but lack descriptive power and subject models of action recognition to extensive training.

In this paper, we propose Natural Action Structures (NASs), i.e., multi-size, multi-scale, spatial-temporal concatenations of local features, as the basic features for representing natural human actions. There are several reasons for using multi-scale representations. First, visual information processing at the retina appears to be multi-scale [20]. Second, the retinotopic map of the visual world has a multi-scale structure, having an expanded central visual field and a compressed peripheral visual field [21]. Finally, the sizes of the receptive fields of the visual neurons increase along the visual pathway [22]. Thus, in this representation of human actions, any action is a spatial and temporal concatenation of a set of NASs, which convey a range of information about natural human actions at spatial-temporal scales. To compile NASs from videos of natural human actions, we first sampled a large number of sequences of patches at multiple spatial-temporal scales. The spatial and temporal scales were so coupled that the sequences at finer spatial scales had shorter durations. We then performed Independent Component Analysis (ICA) [23] on the patch sequences and collapsed the obtained Independent Components (ICs) into clusters using the K-means method. Finally, we obtained a large set of NASs, with each corresponding to a unique combination of the clusters at all the spatial-temporal scales. We examined the statistics of NASs and selected a set of NASs that are highly informative and used them as inputs to two widely used methods for pattern recognition, i.e., Latent Dirichlet Allocation (LDA) and Support Vector Machine (SVM), to classify a range of human actions in the popular KTH and Weizmann datasets. We found that NASs obtained in this way achieved a significantly better recognition performance than simple spatial-temporal features and that the performance was better than or comparable to the best current models. We also found that the recognition performance was slightly degraded by changed spatial-temporal scales and by artificially added noise. These results suggest that NASs can be used as the basic features for encoding human actions and activities. Finally, we discuss how population of neurons might encode NASs and thus human actions and other natural dynamic events.

## Results

### A hierarchical model of natural human actions

**Figure 1:**
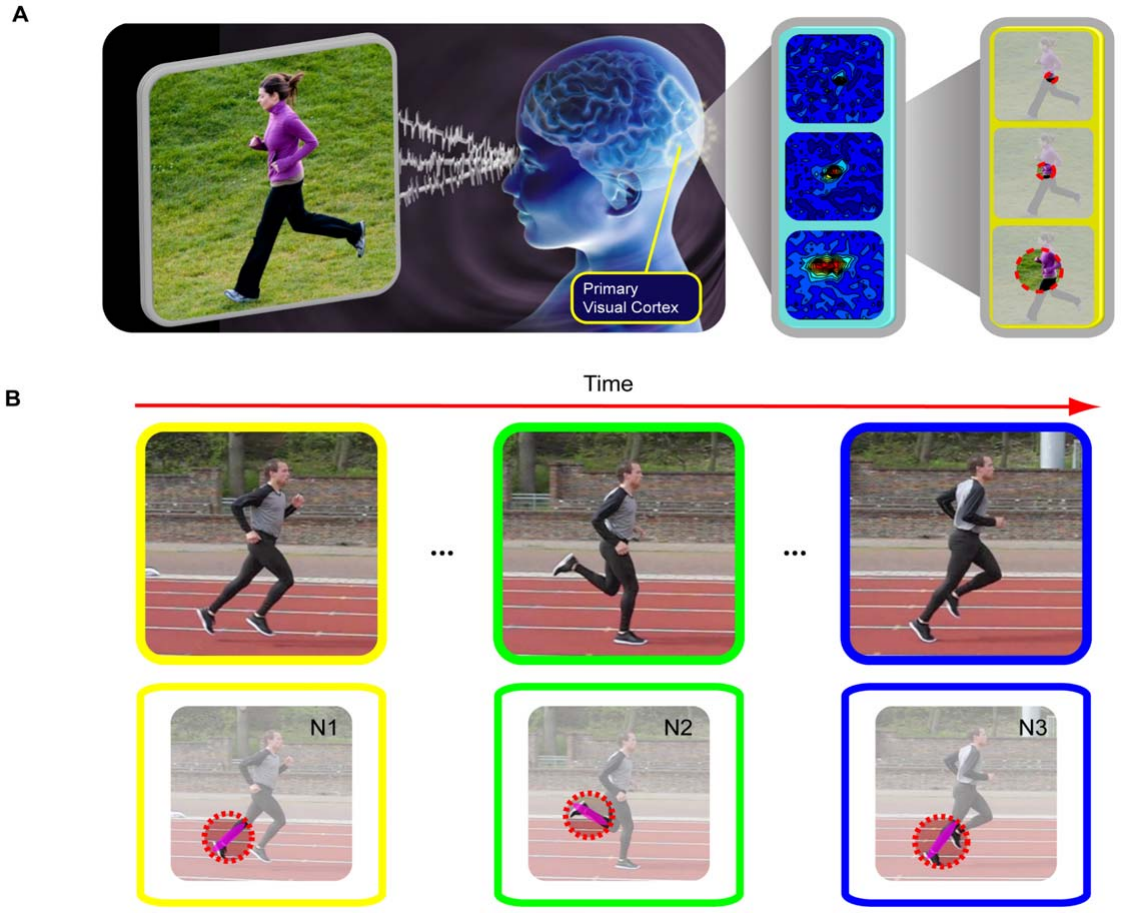
Illustration of the multi-scale neural computation principle in the primary visual cortex. (A), Multi-scale neural computation principle. (B), Illustration of sequential activations of different neurons (N1, N2, and N3) during the action of “running”.

Experimental observations from the primary visual cortex from many animal species suggest that the brain may invariantly deploy an array of neurons with multi-size and multi-scale visual receptive fields (Figure 1 and S1). Accordingly, we have proposed the multi-size and multi-scale coding strategy for computational modeling of rapid scene categorization [24]. We were able to develop a set of encoding units, natural scene structures–multi-scale structured patches in natural scenes, for rapid and accurate scene categorization [24]. We used 70 natural scene structures to encode a variety of animals and 134 natural scene structures to encode a variety of vehicles. With this highly informative structural representation, we can localize and categorize animals in natural scenes and vehicles in street scenes with a near human-level performance. To examine the utility of the multi-size and multi-scale coding strategy for action recognition, here we propose NASs as the basic units for encoding human actions and activities. NASs are multi-size, multi-scale, spatial-temporal concatenations of features in natural actions. Each action is a collection of NASs that are arranged in both space and time domains. In this study, we generalized this multi-scale encoding strategy to: 1) extract all local features at each point of interest in space-time volumes of natural human actions that have sizes significantly greater than those at which usual local features such as gradients and optical flows are computed; 2) compile all combinations of local features in the space-time volumes; 3) apply these two steps to multiple coupled spatial and temporal scales; 4) avoid grouping, segmenting, and tracking that are used to extract features in other approaches; 5) reduce the encoding of natural human actions to a relatively simple spatial-temporal concatenation of a set of encoding units.

We used ICs as local features in space-time volumes of videos of actions at three sizes (i.e., 13 (space)×13 (space)×11 (time), 25×25×21, and 49×49×31), collapsed all combinations of ICs in the space-time volumes into a set of clusters, and assigned all patch sequences that shared the same dominant clusters to a NAS. There are several advantages of using the NASs obtained this way as the basic encoding units of human actions. First, NASs capture structural spatial-temporal information of actions at multiple scales in a compact way. Second, NASs naturally incorporate multi-scale, spatial-temporal features and thus are less sensitive to noises and changes of scales. Finally, NASs provide a simple way to encode natural actions since NASs are local structured descriptions of the space-time volumes of natural actions. In the following sections, we describe how to compile NASs from datasets of videos of human actions, the statistics and the information content of NASs, and our results of action recognition using NASs.

### Compiling natural action structures

**Figure 2:**
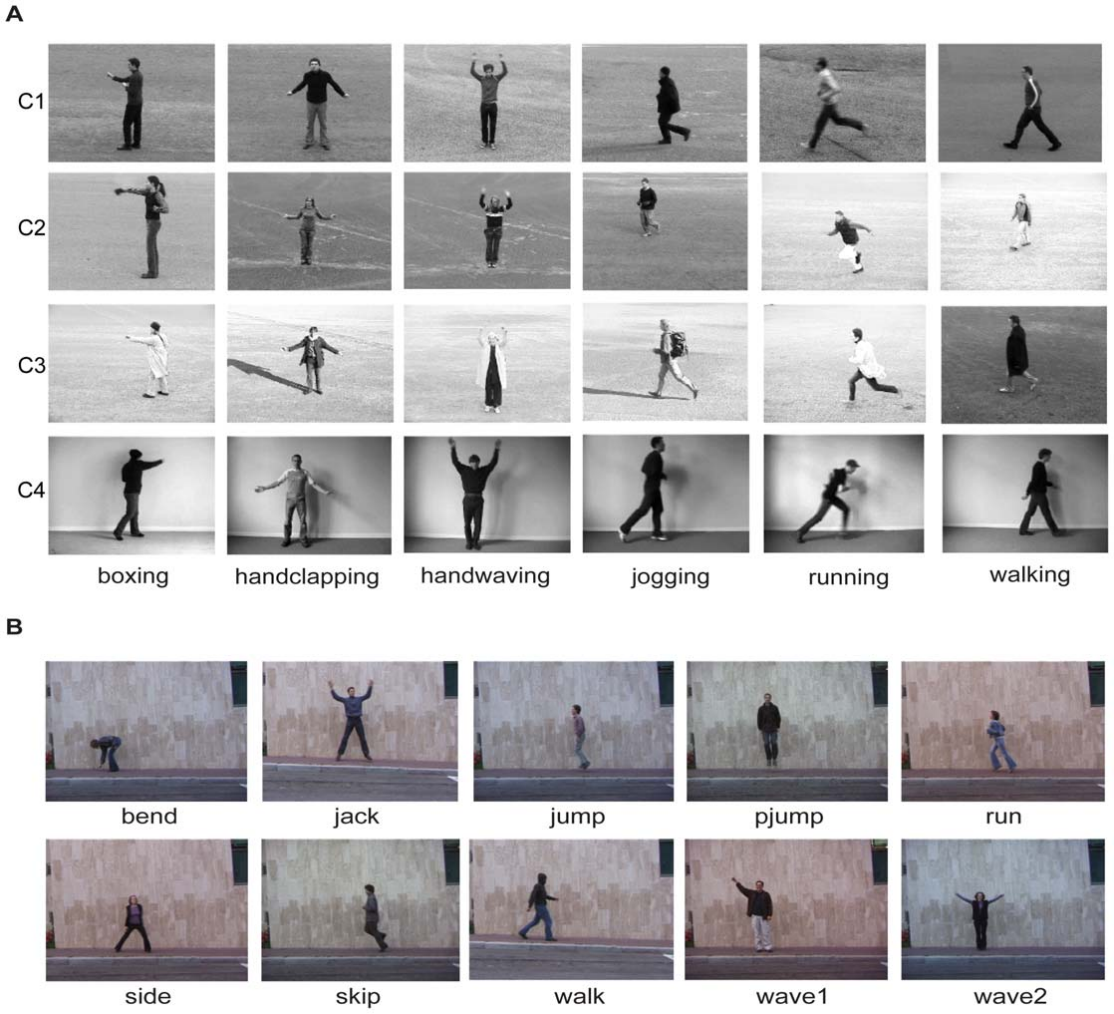
Actions in the KTH and the Weizmann datasets. (A), Six actions in four conditions in the KTH dataset. The four conditions are, from top to bottom, outdoor (C1), outdoor with variations in image scale (C2), outdoor with different cloths (C3), and indoor (C4). (B), Ten actions in the Weizmann dataset.

We took five steps to compile NASs in two widely used video datasets of human actions, the KTH dataset and the Weizmann dataset (see Figure 2 and details in Materials and Methods).

1. Sample a large number of sequences of circular patches at multiple spatial-temporal scales.
2. Perform ICA on the patch sequences at each spatial-temporal scale and obtain ICs at each scale.
3. Fit Gabor functions to the ICs and collapse the ICs into a set of clusters
4. Map the patch sequences to the IC clusters and compute the corresponding feature vectors.
5. Partition the space of feature vectors into a set of structural clusters and obtain the multi-scale NASs.

To generate samples from videos of human actions, we first selected points of interest as the local maxima of the responses of the cuboids detector [17], which is formulated as follows
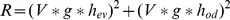
(1)where * denotes convolution operation, *V* is the extracted video volume, *g* is the 2D Gaussian kernel applied to the spatial dimensions, and *h_ev_* and *h_od_* are a quadrature pair of 1D Gabor filters applied to the temporal dimension.

**Figure 3:**
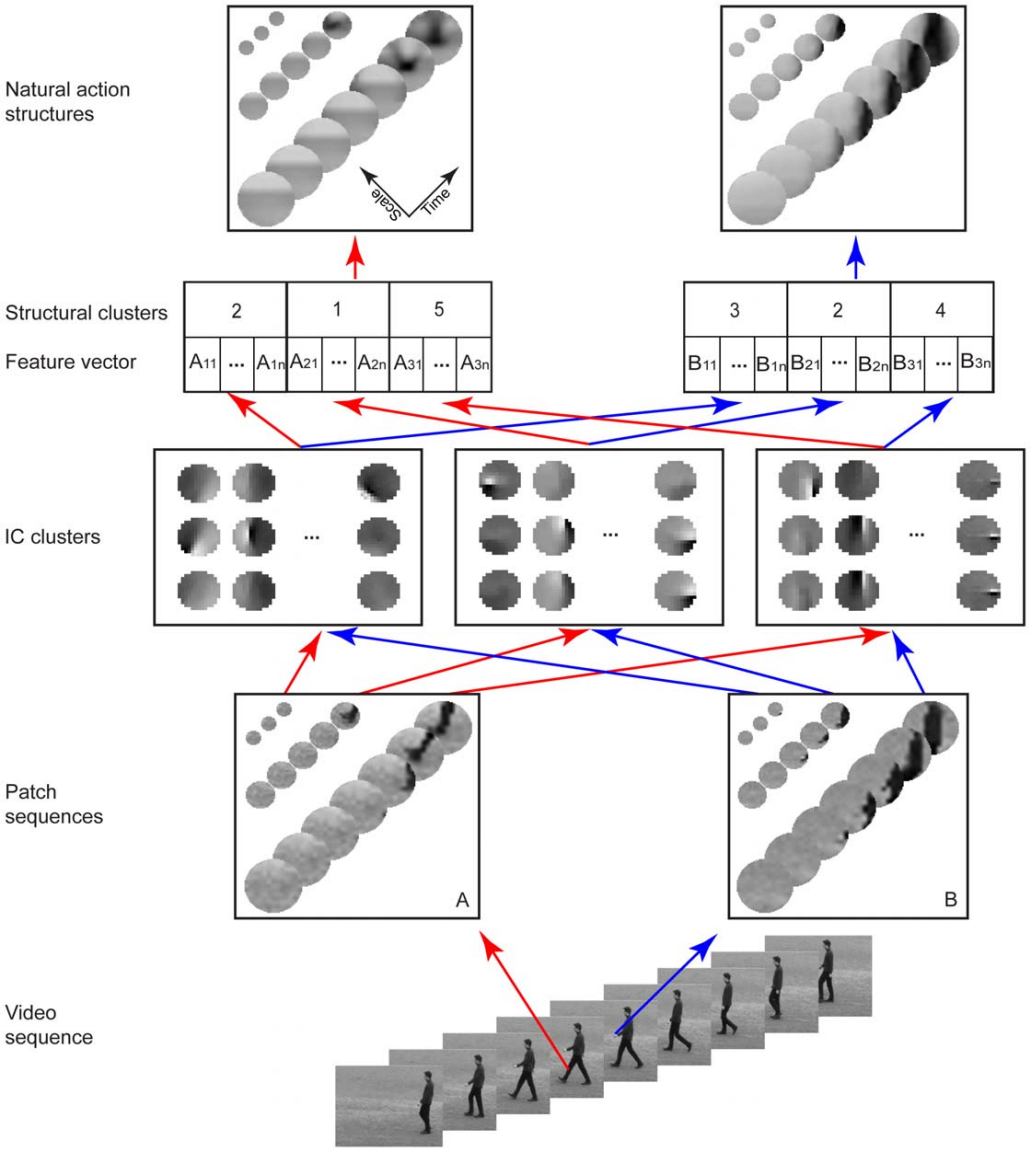
Natural action structures. First, we sampled sequences of circular patches at three coupled spatial-temporal scales centered at selected points of interest from videos of human actions. Second, we performed ICA on the patch sequences, fitted Gabor functions to ICs, and collapsed the ICs into a set of clusters. For each IC cluster, we computed a feature, defined as the root mean square amplitudes of the ICs in the cluster, and obtained a feature space. Finally, we digitized the feature space into a set of non-overlap regions using the K-means method, assigned an index to each region. We applied this procedure at the three spatial-temporal scales separately, concatenated the three indices, and designated all patch sequences that shared the same indices as a NAS. Note that each NAS is a set of patch sequences and also that each NAS shown is the average of all patch sequences that share the same structural indices. For illustration purposes, the numbers of video frames are down-sampled to 3, 5, and 7 for the three spatial-temporal scales.

At the selected points of interest, we sampled sequences of circular patches using the configuration shown in Figure 3. Each circle is a circular patch and the configuration has 3 temporal scales (i.e., 11, 21, and 31 video frames) and 3 spatial scales (i.e., the diameters of the circles are 13, 25, and 49 pixels). The spatial and temporal scales are coupled so that the sequences at finer scales have shorter durations. The rationale for using this configuration is to capture dynamic structures at multiple spatial-temporal scales to achieve robustness, which will become clear later on. To make computing more efficient, we down sampled the sequences at larger spatial-temporal scales. For example, we reduced the patch sequences of 25×25×21 at the middle scale to sequences of 13×13×11 by picking up the frames or pixels indexed by even numbers starting from the center of the sequences along both the spatial and temporal dimensions. Thus, all the patch sequences had the same number of frames and the same patch size.

**Figure 4:**
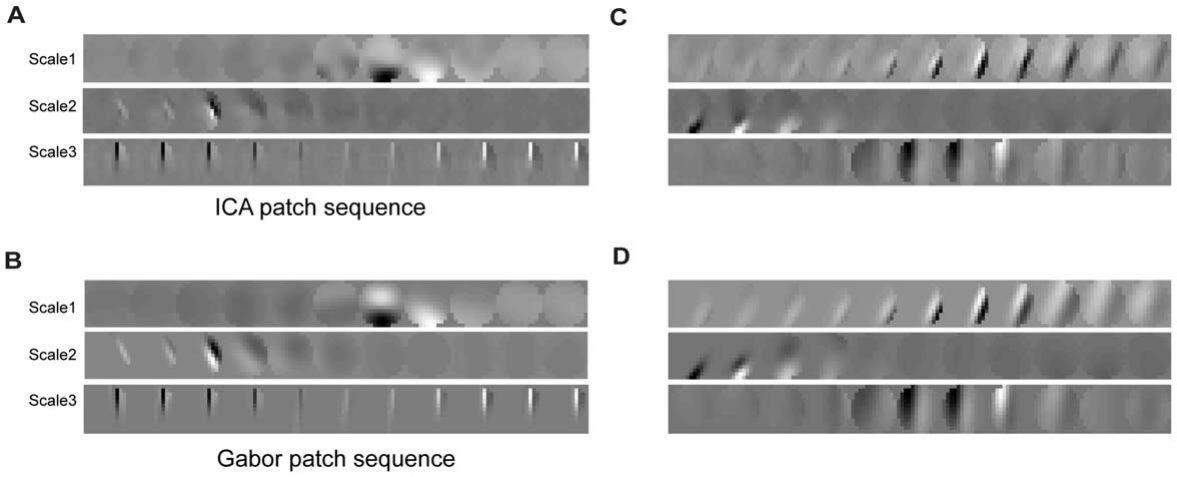
Fitting Gabor functions to ICs. (A), ICs of the three patch sequences sampled at three scales (Scale1, Scale2, and Scale3) from the KTH dataset. (B), fitted Gabor functions of the ICs shown in A. (C) and (D) are the same as (A) and (B) respectively for the Weizmann dataset. Note that, in each figure from top to bottom, the actual sizes of patch sequences are increased and the sequences at larger sizes are down-sampled.

We then performed ICA on the patch sequences at the three spatial-temporal scales and fitted the Gabor functions to ICs. The fitting algorithm worked well, accounting for 85% the variance of the ICs. Figure 4 shows a few examples. In these examples, as expected, the sequences of the ICs entail appearance, disappearance, rotation, translation, and phase shift of oriented bars, all of which are well captured by the fitted Gabor functions.

To derive a compact representation of the ICs obtained above, we performed clustering in the parameter space of the fitted Gabor functions. For this purpose, we used 6 parameters of the Gabor functions, i.e., 4 parameters for the scale and location of the Gaussian envelope and 2 parameters for the orientation and the phase of the sinusoid carrier. Since different values of the parameters may correspond to the same Gabor function (e.g., adding 2π to the phase does not change Gabor function), we converted the estimated parameters to pre-set intervals (see details in Materials and Methods). We then performed clustering using the K-means method with the Euclidean distance metric. Let *A* = {*A*_1_, *A*_2_, …, *A_m_*} denote the *i*-th IC cluster containing *m* filters at a spatial-temporal scale, each of which is a column vector with *l* elements, where *l* is the number of pixels in each patch sequence. Then the feature, *a_i_*, of a patch sequence *P* (which is a row vector with *l* elements) projected to the *i*-th IC cluster is calculated as follows
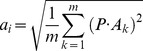
(2)Thus, for *N* IC clusters, there are *N* features which form a feature vector at the spatial-temporal scale. Since the patch sequences sampled from a set of actions do not uniformly pack the high-dimensional feature space, we need to partition the feature space into discrete blocks. To this end, at each spatial-temporal scale, we classified the feature vectors into a set of clusters (referred to as structural clusters) using the K-means method with the Euclidean distance metric. We call all the patch sequences that share the same structural clusters at the three spatial-temporal scales a NAS. Note that, for illustration purpose only, each of the NASs shown in the figures is the average of the patch sequences corresponding to that NAS.

**Figure 5:**
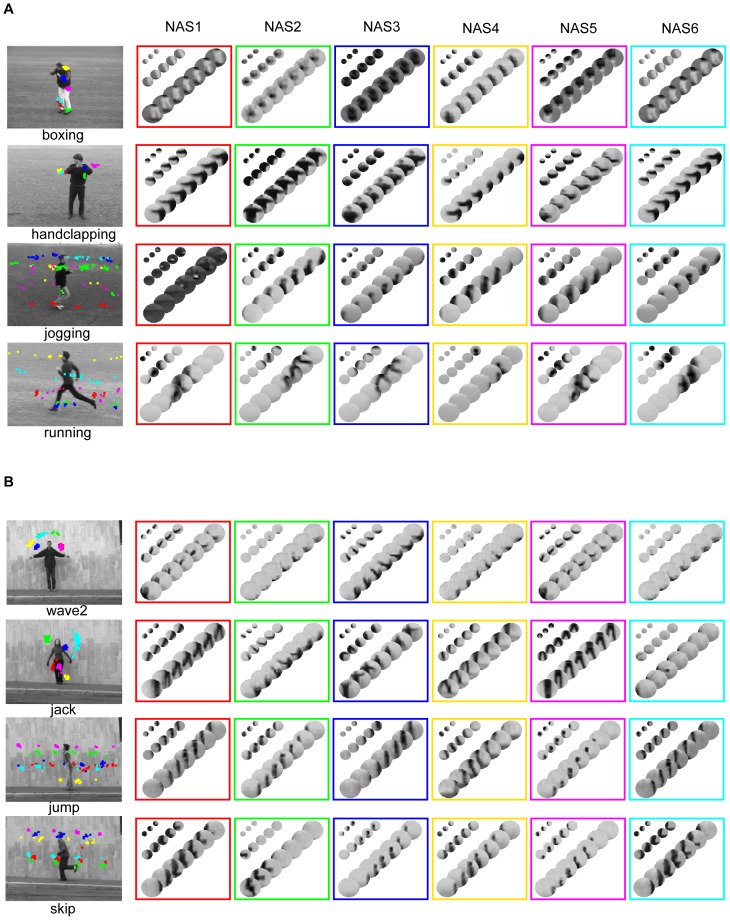
Examples of natural action structures. (A), 6 frequent NASs compiled from each of the 4 actions in the KTH dataset. The locations of the NASs in the videos and the NASs are indicated by the same color. (B), Same format as (A). 6 frequent NASs compiled from each of the 4 actions in the Weizmann dataset. Note that each NAS is a set of patch sequences and also that each NAS shown is the average of all patch sequences that share the same structural indices.

In contrast to simple features, such as ICs and cuboids descriptors and simple spatial-temporal statistics (e.g., histogram of optical flow in a small region), NASs are highly structured patch sequences that are building blocks of natural actions. Roughly speaking, as a result of the K-means clustering procedures, instances of any NAS are patch sequences cropped from videos of human actions that share the same structural indices described above and the NASs for any natural human action can range from simple to complex spatial-temporal concatenations of local features. Figure 5A shows 6 most frequent NASs of each of the four selected actions in the KTH dataset. These structures represent coarse but informative descriptions of a variety of movements of body parts. For example, in the boxing action, the six most frequent action structures (indicated by six different colors) describe, respectively, the movements of the right leg, the left foot, the left arm, the left shoulder, the waist, and the right leg. In the jogging action, the six most frequent action structures describe, respectively, the movements of the foot, the shoulder, the head, the shoulder, the body, and the head. Figure 5B shows 6 most frequent NASs for each of the four selected actions in the Weizmann dataset. As with the KTH dataset, these structures are informative descriptions of various parts of the body movements. For example, in the wave2 action, the six most frequent action structures describe, respectively, the movements of the left arm, the right hand, the right arm, the right arm, the left arm, and the right hand. In the jump action, the six most frequent action structures describe, respectively, the movements of the waist, the shoulder, the waist, the foot, the head, and the waist.

### Statistics of natural action structures

**Figure 6:**
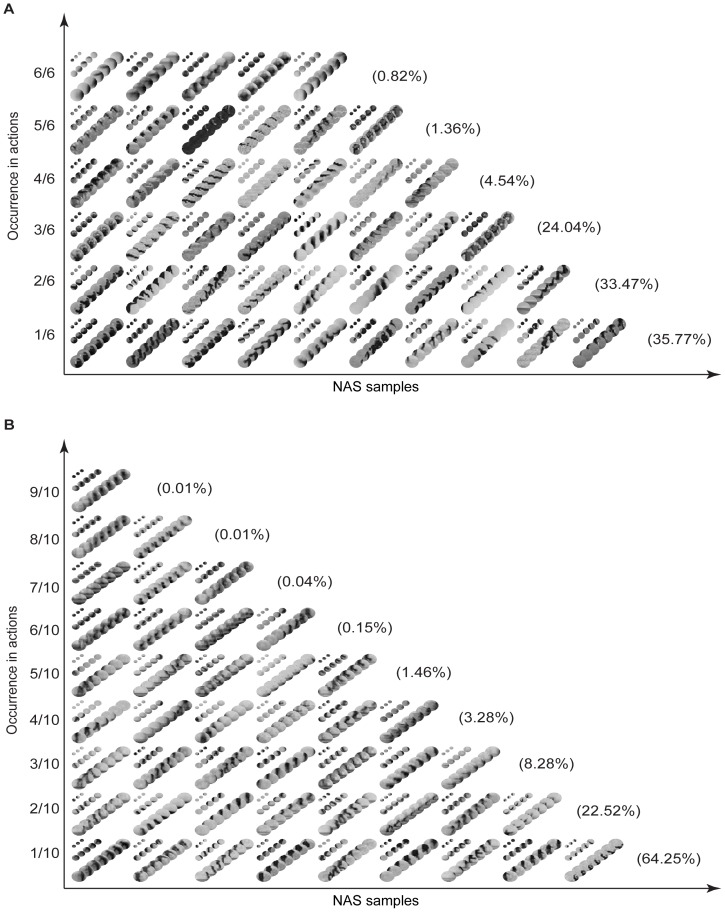
Statistics of occurrences of NASs. (A), Examples of NASs shared by 1 to 6 actions in the KTH dataset. The numbers are the percentages of the NASs shared by 1 to 6 actions. (B), Same format as (A) for the Weizmann dataset. Note that each NAS is a set of patch sequences and also that each NAS shown is the average of all patch sequences that share the same structural indices.

We obtained 193,600 NASs in the KTH dataset and 11,540 NASs in the Weizmann dataset. As shown in Figure 6, NASs shared by more actions tend to be blurry while NASs occurring in only one or two actions are usually coarse local movements in the actions. To use NASs to categorize human actions, we need a procedure to select informative NASs. We selected NASs for action classification in two steps. In the first step, we selected NASs that occurred more than Mc times in the training set to exclude rarely occurring NASs. In the second step, we used a One-vs.-One strategy to select discriminative NASs based on the histograms of the NASs. For each pair of actions, we selected the first Nc most discriminative NASs of each action by comparing the histograms of the NASs in the two actions. Take boxing vs. handclapping as an example. For the boxing action, we selected the Nc NASs that had the smallest occurring frequency in the handclapping action and the biggest difference in occurring frequency in the two actions. We defined these Nc NASs as the most discriminative NASs for boxing in boxing vs. handclapping. We then applied the same procedure to the handclapping action. To set the thresholds, Mc and Nc, we used a procedure called Leave-One-Out Cross-Validation (LOOCV) (see definition in Material and Methods). As a result, we selected 4,998 NASs in the KTH dataset and 6,392 NASs in the Weizmann dataset.

Next, we examined the statistics of the NASs, which include: 1) the occurring frequencies of NASs, 2) the correlation between NASs, and 3) the information content of NASs, defined as One-vs.-Rest classification performance for each action. Figure 6 shows the statistics of occurrence of NASs in the two datasets. Simpler NASs (e.g., the NASs in the first row (6/6) of Figure 6A) occur more frequently and are shared by more actions, and most NASs are shared by only a few actions. The percentages of the NASs that are shared by 1 to 6 actions in the KTH dataset are 35.77%, 33.47%, 20.04%, 4.54%, 1.36%, and 0.82% respectively (Figure 6A). The percentages of the NASs that are shared by 1 to 9 actions in the Weizmann dataset are 64.25%, 22.52%, 8.28%, 3.28%, 1.46%, 0.15%, 0.04%, 0.01%, and 0.01% respectively (Figure 6B). Thus, in both datasets, only a few percent of the NASs are shared by more than three actions. NASs shared by most of the actions contain few structures and are blurry. For example, the third NAS in the first row (6/6) of Figure 6A is shared by all the actions, but conveys little information about any of the actions. On the other hand, NASs shared by only one or two actions contain more structures and more action-specific information. For example, in Figure 6A, the fourth NAS in the fifth row (2/6) occurs in handclapping and jogging classes and describes arm movements and leg movements in the two actions, respectively. In Figure 6B, the NAS in the first row (9/10) is shared by all the actions except run. We can see a blurry body shape in it. The fourth NAS in the seventh row (3/10) is shared by jump, skip, and walk and describes body movements shared by the three actions.

**Figure 7:**
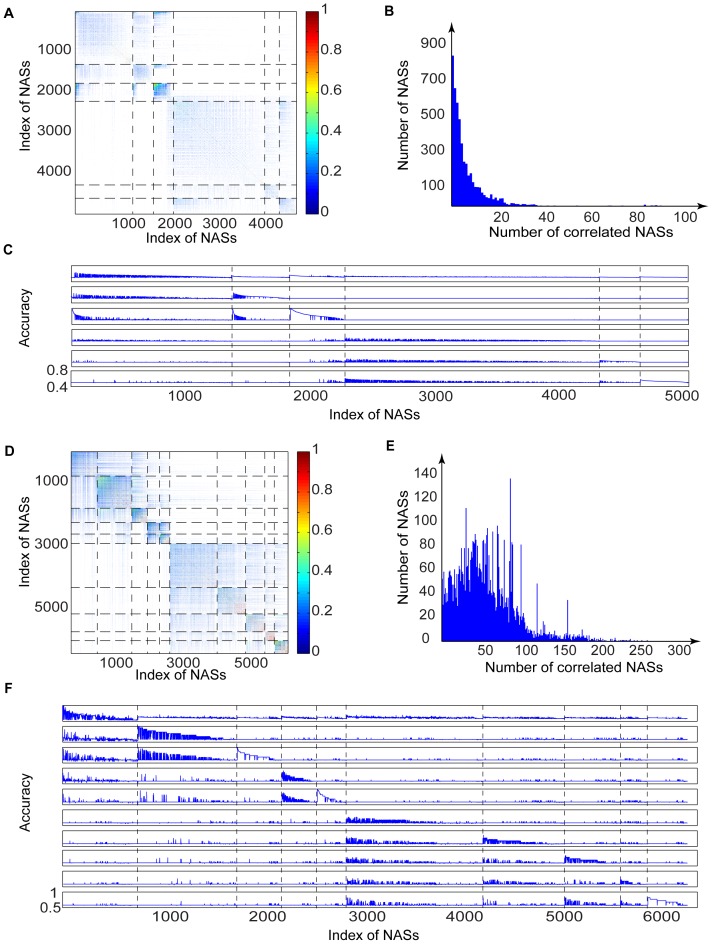
Statistics of NASs. (A), Correlation coefficients between NASs. (B), The number of NASs that are correlated with (correlation coefficient > = 0.5) a certain number of other NASs. (C), Each curve indicates the One-vs.-Rest classification accuracy for each action. In (A), NASs are indexed sequentially by the occurrences in boxing, handclapping, handwaving, jogging, running, and walking. (A–C) are for the KTH dataset. (D–F), Same format as (A–C) for the Weizmann dataset. In (F), structures are indexed sequentially by the occurrences in bend, jack, pjump, wave1, wave2, jump, run, side, skip, and walk.

Figure 7A shows the correlation matrix of the NASs in the KTH dataset. The dashed lines indicate the ranges of the indices of the NASs complied from each action. The indices are so arranged that no two or more NASs share the same index. There are two clusters in the matrix in the upper left corner and the lower right corner. These clusters indicate that NASs in the in-position actions (i.e., boxing, handclapping, and handwaving) tend to be correlated with each other and have weak correlation with NASs in the moving actions (i.e., running, jogging, and walking). There is also stronger correlation among NASs in the same action. Figure 7B shows the numbers of NASs that are correlated with certain numbers of other NASs (correlation coefficients greater than 0.5). Most of the NASs are correlated with only a few other NASs. Roughly, 100 of the 4,998 NASs are correlated with 10 NASs and only 40 are correlated with 20 NASs. Thus, NASs compiled from this dataset are quite sparse.

To examine the information content of NASs, we developed a Gaussian model of the occurring frequency in video samples of each action for each NAS, and classified the actions based on models via Bayesian inference. We then performed LOOCV on the training dataset, and sorted the NASs in each action according to the One-vs.-Rest classification accuracy. Figure 7C shows the One-vs.-Rest classification accuracy for each NAS, which is indexed the same way as Figure 7A. As shown in Figure 7C, each action has a set of NASs with high discriminative information; some NASs convey information about multiple actions; most NASs convey information about one action; and NASs in the in-position actions contain higher discriminative information than the moving actions.

We also obtained the similar results on the Weizmann dataset, which are shown in Figure 7D–E. Figure 7D shows that the NASs in the Weizmann dataset have higher correlations within the same actions than in the KTH dataset since the Weizmann dataset has relatively small variations. Also, more NASs in this dataset are strongly correlated with other NASs (Figure 7E). Figure 7F shows the One-vs.-Rest accuracy for the ten actions in the Weizmann dataset. Compared to the NASs in the KTH dataset, NASs in the Weizmann dataset have higher information content.

**Figure 8:**
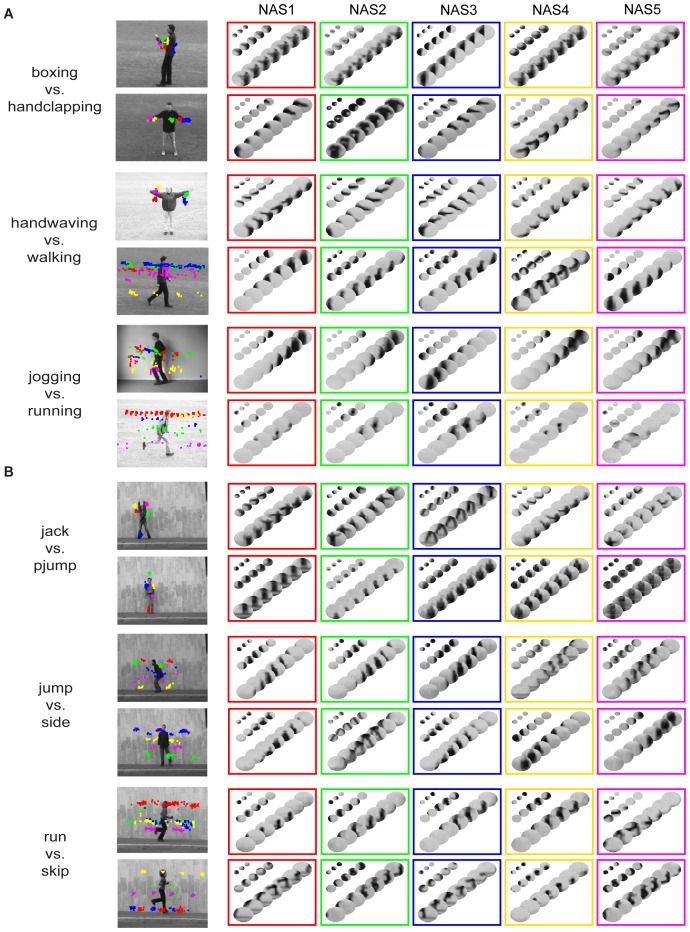
One-vs.-One discriminative natural action structures. (A), One-vs.-One discriminative NASs in three pairs of actions in the KTH dataset. The locations and NASs in each individual action are indicated by the same color. (B), Same format as (A) for the Weizmann dataset. Note that each NAS is a set of patch sequences and also that each NAS shown is the average of all patch sequences that share the same structural indices.

Another way to examine NASs is to find the NASs that best discriminate pairs of actions as described above. Figure 8 shows 10 NASs for each of six selected pairs of actions. For boxing vs. handclapping, NASs describing arm movements are the best discriminative features. For handwaving vs. walking, NASs describing arm movements and upper-body movements are the best discriminative features. For jogging vs. running, NASs describing lower-body and leg movements and NASs describing head, upper-body, and leg movements are the best discriminative features. For jack vs. pjump, NASs describing arm and leg movements and NASs describing head, body, and foot movements are the best discriminative features. For jump vs. side, NASs describing head, body and leg movements and NASs describing body and leg movements are the best discriminative features. The most similar pair of actions in the Weizmann dataset is run and skip. For the run action, the discriminative NASs describe head, body and leg movements and, for the skip action, the discriminative NASs describe head, body and foot movements.

Thus, NASs contain a full range of concatenations of spatial-temporal features within space-time volumes with a size of 49 (space)×49 (space)×31 (time) and convey a range of information about human actions. In the next section, we show how to use NASs for action recognition.

### Action classification

We used NASs as inputs to two methods for pattern classification, LDA and SVM. LDA is a hierarchical probabilistic model [25]. It has been used in several applications [26]–[28] in computer vision. SVM is a discriminative model, which seeks the optimal decision boundary between classes directly [29]. It is of interest to compare these two classifiers as applied to action recognition.

**Figure 9:**
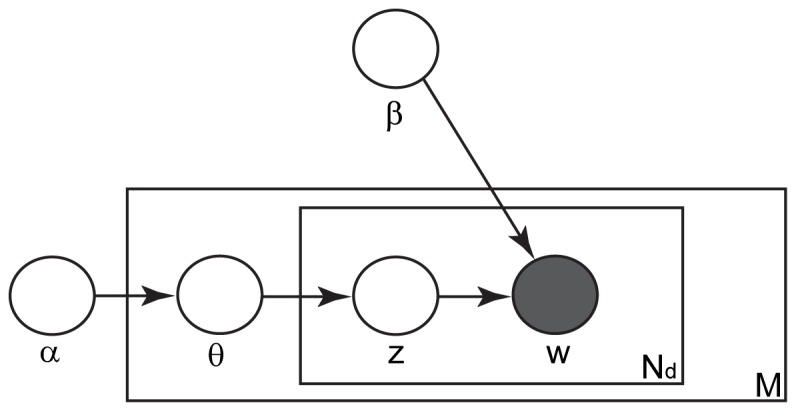
Latent Dirichlet Allocation model. The nodes represent random variables. The shaded nodes are observed variables and the unshaded nodes indicate unobserved variables. The plates indicate repetitions. *M* is the number of video sequences of each action, *N_d_* is the number of code words *w* in the *d*-th video sequence, and *z* is the hidden topic. The parameters of the model include *α*, the parameter of the Dirichlet distribution, *θ*, the parameter of the multinomial distribution, and *β*, which parameterizes the multinomial distribution conditioned on topic *z*.

In LDA, the probability distributions (PDs) of the code words (i.e., features) for each class are estimated and classification is performed via Bayesian inference. Suppose that we have *M* video sequences of each action video set *D* and each video sequence contains *N* spatial-temporal code words *w* = {*w*_1_, *w*_2_, …, *w_N_*}, a subset of a code book of size *C*. Each video sequence can be seen as generated via the following process (Figure 9).

1. Choose *N*∼Poisson(*ξ*)
2. Choose *θ*∼Dirichlet(*α*)
3. For each of *N* words *w_n_*
  - Choose a topic *z_n_*∼Multinomial(*θ*)
  - Choose a word *w_n_* from multinomial distribution *p*(*w_n_*|*z_n_*, *β*) conditioned on topic *z_n_*

*ξ* is the mean of Poisson distribution, Poisson(*ξ*), *α* is the parameter of *K*-dimensional Dirichlet distribution Dirichlet(*α*) (*K* is the number of hidden topics *z_n_*), and *θ* is the parameter of multinomial distribution Multinomial(*θ*), which determines how the *K* topics are mixed in the current video sequence. Given topic *z_n_*, code word *w_n_* is generated according to *p*(*w_n_*|*z_n_*, *β*), a multinomial distribution parameterized by *β*, a *K*×*C* matrix. Thus, under the i.i.d. (independent and identically distributed) assumption, the LDA model for an action video set *D* is
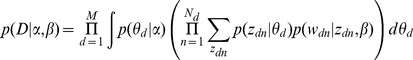
(3)where *d* is the index of video sequences and *n* is the index of code word. Since it is difficult to estimate the parameters in this model directly, as proposed in [25], we used the following variational distribution to approximate the posterior PD of hidden variables, 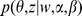, and learn the LDA model by using the variational Bayesian method.
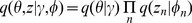
(4)where *γ* and *φ* are the variational parameters and *n* is the index of code words.

By design, LDA is a bag-of-words model and leaves out spatial and temporal interactions among NASs, which will be discussed later. Several extensions have been proposed to address this limitation [30]–[32].

In this work, NASs are used as code words and spatial-temporal concatenations of NASs as topics. Thus, by definitions, parameter *θ_d_* stands for the mixing weight of the concatenations of NASs for each video sample *d*, *α* controls the distribution of each *θ_d_*, and *β* defines the distribution of NASs in each topic *z*. To test this model, we developed a LDA model for each action and performed classification via Bayesian inference.

SVM is another widely used classifier [14], [18], [33], [34], which we describe briefly by taking binary classification as an example. In this case, the data format is 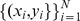, where 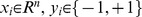, and the task is to find the best hyperplane in the feature space *F* that separates the two classes by optimizing
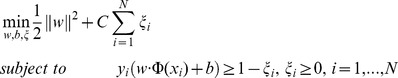
(5)

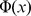
 maps data vector *x* into the feature space *F*, *w* is a weight vector, *b* is a bias, *ξ* are slack variables, and *C*>0 is a regularization constant. By introducing Lagrange multipliers *α_i_*, we obtain the dual problem
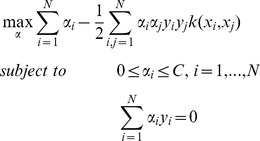
(6)where 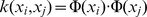 is a kernel function. The decision function is as follows
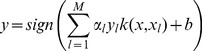
(7)where *M* is the number of non-zero *α* and *l* is the index of non-zero *α*.

In this work, we used 1-*χ* ^2^ kernel [35], [36].
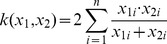
(8)where *x*_1*i*_ is the *i*-th element of *x*_1_. To use this formulation for multi-class action recognition, we used the One-vs.-Rest strategy and LIBSVM implementation of SVM [37], [38]. To incorporate spatial information into the SVM classifier, inspired by [18], we partitioned spatial and temporal dimensions of the video sequences into n-m-k grids (i.e., n equal grids in vertical dimension, m equal grids in horizontal dimension, and k equal grids in temporal dimension), concatenated the histograms of NASs in the grids into a big feature vector, and used the pooled histograms as inputs to the SVM model.

We compared the performance of the two classifiers with NASs as inputs on the KTH and the Weizmann datasets to the performance of two baseline methods (Table 1). In the baseline methods, we used the cuboids descriptors as inputs to the LDA model and the SVM classifier described above. For the LDA classifier with NASs as inputs, the recognition rate is 89.5% on the KTH dataset and 94.4% on the Weizmann dataset. For the SVM classifier with NASs as inputs, the recognition rate is 92.7% on the KTH dataset and 96.7% on the Weizmann dataset. For both classifiers, using NASs as inputs improves recognition performance. The error reduction rate relative to the performance of the cuboids model (i.e., the ratio of improvement to the error in the cuboids model) is up to 37% on the KTH dataset and up to 41% on the Weizmann dataset.

**Table 1:** Performance of the proposed models and the baseline models.

Methods	KTH	Weizmann
**Cuboids+LDA**	85.0%	90.0%
**Cuboids+SVM**	88.5%	94.4%
**NASs+LDA**	89.5%	94.4%
**NASs+SVM**	92.7%	96.7%

These results also show that the SVM model achieves better performance than LDA consistently. There are two reasons. First, SVM seeks a decision surface with the maximal margin of separation, which is usually beneficial for classification with a large number of features and relatively small number of training samples. Second, we concatenated the histograms of NASs in multiple partitions of the video sequences in our implantation of SVM, but we did not model any spatial-temporal interactions among NASs in LDA.

**Figure 10:**
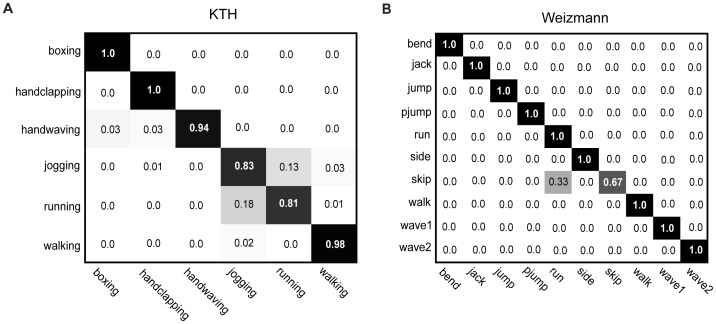
Confusion matrices. (A), Confusion matrix of the performance of the SVM with NASs on the KTH dataset. The average accuracy is 92.7%. (B), Confusion matrix of the performance of the SVM with NASs on the Weizmann dataset. The average accuracy is 96.7%.

Figure 10 shows the confusion matrices of the performance of the SVM model on the KTH dataset and the Weizmann dataset. For the KTH dataset, the in-position actions and the moving actions can be well classified, while ∼16% of the incidences of jogging and running were misclassified. On the Weizmann dataset, our model only misclassifies 33% of the incidences of skipping as running. These two actions are indeed very similar.

We compared our model with the best current methods (Table 2). For the KTH dataset, we used the training-testing separation coming with the dataset, the same scheme used in [18], [39]. In the other models cited in the table [27], [40]–[42], LOOCV was used to obtain the performance (in [42], the authors used 20-fold cross validation based on randomly selected training and testing datasets), which is usually better than performance obtained without cross-validation. For the Weizmann dataset, because of the limited samples, all the results were obtained using LOOCV. It should be also noted that the 100% performance on the Weizmann dataset was achieved by using global space-time shape features segmented from background. Based on these comparisons, we concluded that the performance of the SVM classifier with NASs as features on both datasets of human actions is among the best current models.

**Table 2:** Performance of the proposed models and the state-of-the-art models.

Methods	KTH	Weizmann
**Proposed Method (SVM)**	92.7%	96.7%
**Wang et al. (2011) ** [**39**]	92.5%	-------
**Yao et al. (2010) ** [**37**]	92.0%	95.6%
**Wang et al. (2009) ** [**36**]	92.1%	-------
**Bregonzio et al. (2009) ** [**38**]	93.2%	96.7%
**Laptev et al. (2008) ** [**13**]	91.8%	-------
**Niebles et al. (2008) ** [**24**]	83.3%	90.0%
**Gorelick et al. (2007) ** [**11**]	-------	100.0%

**Figure 11:**
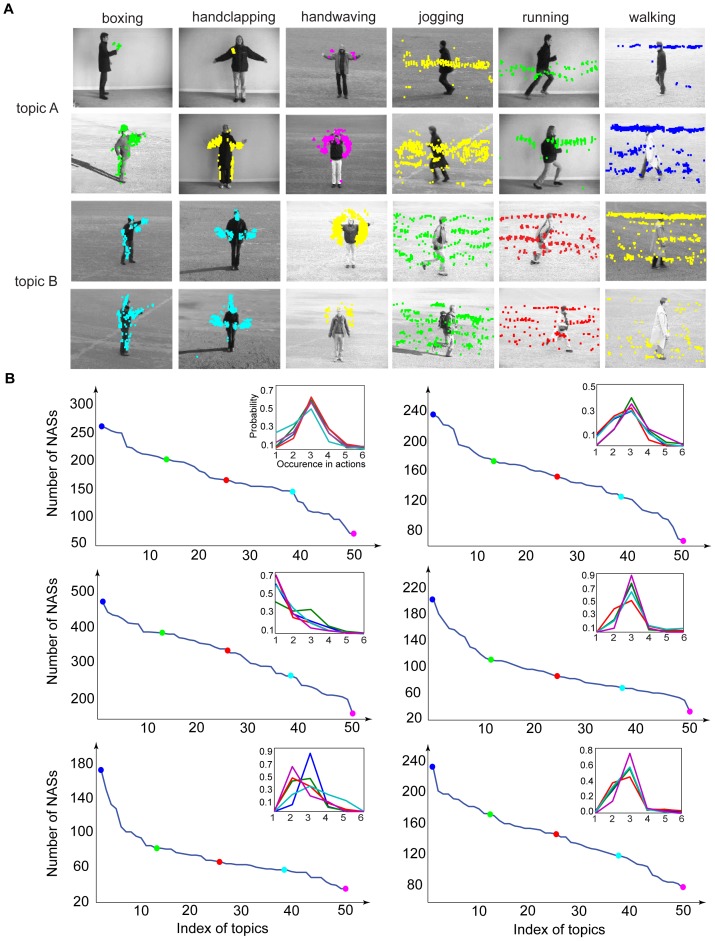
Action topics in LDA. (A), Locations of the NASs that share the same topic in the LDA model. Two topics are shown for each action in the KTH dataset. (B), Number of NASs share the same topic of the LDA models of the six actions in the KTH dataset. The insets show the numbers of NASs of the selected topics that are shared by the 1–6 actions in the dataset. Note that, by definition, topics are not shared by two or more actions.

We also examined the topics of the LDA models of the actions in the KTH dataset. In LDA, topics are on a higher hierarchy than code words, which can be seen as being sampled from PDs conditioned on topics (Figure 9). Since we compiled NASs and used them as code words, it is of interest to examine the topics in these models. We developed a LDA model for each action and signed NASs to a topic z according to the following equation
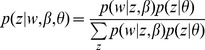
(9)We obtained 50 topics for each action in the KTH dataset. Figure 11A shows two topics A and B (indicated by different colors) in 4 examples of each of the 6 actions in the KTH dataset. Although code words (i.e., NASs) usually describe specific movements of body parts (Figure 5), topics are much less specific in terms of body parts and/or movements. For example, in the boxing action, topic A can be hand movement (top panel) or movements of head, arms, and legs (middle panel). In another example, topic B covers almost all the body movements in the boxing action. In the running action, topic A can be leg movements (top panel) or upper-body movements (middle panel); topic B covers almost all the body movements in the action. Thus, there are no fixed spatial positions of the topics in the LDA model. The underlying reason is that spatial and temporal relationships among code words are ignored in LDA and that there are no other constraints on topics. Figure 11B shows the numbers of NASs sharing the same topic of the LDA models. Each inset shows the relative occurring frequencies of the NASs of the selected topics that are shared by the 1–6 actions in the KTH dataset. A main feature of the occurrences of NASs of the topics is that the NASs of each selected topic are usually shared by two or three actions, except for handwaving, where most NASs of each selected topic occur mainly in handwaving. Another feature is that in each action the curves of the relative occurring frequencies are more or less the same while the number of the NASs of each topic varies significantly.

**Figure 12:**
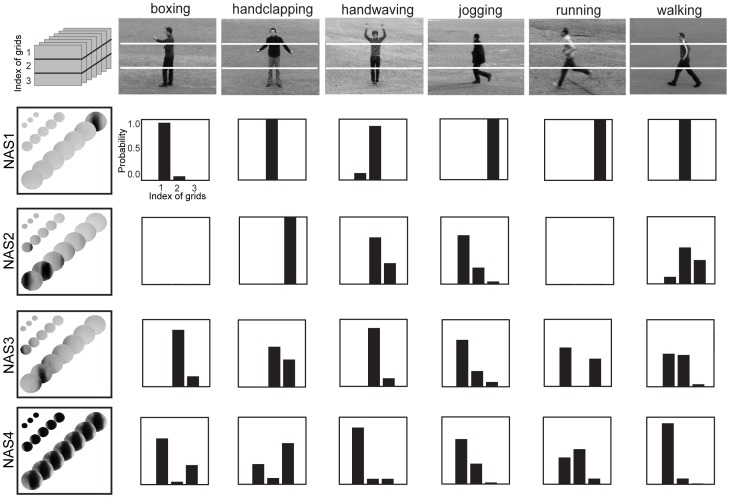
Spatial distributions of NASs. Spatial distributions of NASs in the 6 actions in the KTH dataset. The panels show the normalized frequencies of the NASs shown to the left, in the three areas indicated by the numbers on the video frame. The top-left figure shows the partition grids. Note that each NAS is a set of patch sequences and also that each NAS shown is the average of all patch sequences that share the same structural indices.

While we did not consider spatial and temporal interactions among code words in our implementation of LDA, we did test a simple way to deal with these interactions in our implementation of SVM on the KTH dataset as described above. The performance was improved from 91.4% to 92.7% by using the pooled features in 3-1-1 grids as illustrated in top-left of Figure 12. This improvement arises from the fact that the spatial distributions of NASs may be different for different actions. Figure 12 shows the distributions of 4 NASs in the three parts in the 6 actions. Take the first NAS as an example. Most samples of the NAS occur in the upper grid in the boxing action, occur in the middle grid in the handclapping, handwaving, and walking actions, and occur in the lower grid in the jogging and running actions.

Finally, we examined how the recognition performance on the KTH dataset would be affected by variations in scale and noise. For scale variations, we generated two test datasets, ScaleP10 and ScaleN10. In ScaleP10, we increased the sizes of video frames by 10% along both vertical and horizontal directions. In ScaleN10, we decreased the sizes of video frames by 10%. The classification accuracies of the SVM model on ScaleP10 and ScaleN10 were 88.1% and 90.7%, respectively. We also added white Gaussian noise at each pixel of the video frames with a signal-to-noise ratio of 20 db (i.e., Std_noise_/Std_signal_ = 0.1) on both the training and testing datasets, and retrained the SVM classifier. The classification accuracy on the noise dataset was 91.4%.

As a final note, we should mention several reasons why the large number of NASs does not impair the generalization of the proposed methods. First, as described above, we selected the most discriminative NASs for action recognition. Second, we randomly selected a small portion of the training data (11.9% for the KTH dataset and 17.5% for the Weizmann dataset) for compiling NASs. Thus, the extracted NASs did not tend to over-fit the training set. Finally, some NASs encode similar information, i.e., there is redundancy among NASs.

We implemented the proposed procedure in Matlab (Version 7.8.0.347) running on a Dell Optiplex 960 desktop (with an Intel E8500 3.16 GHz processor and 8G RAM). On average, it took 43.9 s to categorize each video of 100 frames. The main load was to compute NASs from input videos. This step can be greatly speeded up by using more efficient searching algorithms.

## Discussion

### Natural action structures

In this study we proposed NASs as the basic spatial-temporal features for action recognition based on a range of evidence for multi-scale information processing in the visual system [20]–[22]. Under this concept, a NAS is a concatenation of sequences of structured patches at multiple spatial-temporal scales and each sequence of structured patches is a concatenation of a set of dominant ICs of natural human actions. To make NASs more compact while maintaining their descriptive power, we proposed to combine ICs of natural human actions into a set of IC clusters, to use the IC clusters to characterize sequences of structured patches, and to classify sequences of structured patches into a set of structural clusters. There are several advantages of using NASs. First, compiling of NASs requires, in principle, no isolation of objects or figure-background segmentation, nor computing of global spatial-temporal features. Second, NASs are not sensitive to noises and changes in scales since they are concatenations of local features at multiple spatial-temporal scales. Third, NASs contain a variety of multi-scale information about human actions; general structures are shared by more actions while specific structures are shared by only one or two actions. Finally, encoding of actions by NASs is more or less equivalent to specifying the spatial and temporal arrangements of NASs, which reduces the training of models for recognition.

We used NASs as the basic features for action recognition in two popular recognition methods, LDA and SVM, on two datasets of human actions. For LDA, the recognition rate is 89.5% on the KTH dataset and 94.4% on the Weizmann dataset. For SVM, the recognition rate is 92.7% on the KTH dataset and 96.7% on the Weizmann dataset. For both methods, these results are better than those obtained using low-level features and are among the best current models of action recognition. We also found that the classification performance with NASs as features was slightly affected by changes of scale and noise.

### Possible neural mechanisms

Neurons in the primary visual cortex respond selectively to stimulus orientations, spatial frequency, speed, and directions [43], [44]. By the time information reaches neurons in the MT area, local visual motion signals are integrated into the global motion of complex objects [45]. Recent studies [46], [47] showed that the responses of V1 neurons can be described to a large degree by the Linear-Nonlinear (LN) model where the responses of a set of linear filters are combined in a simple nonlinear way. Furthermore, the responses of MT neurons can be described by a nonlinear function of a weighted sum of responses of V1 neurons [48]. These observations strongly suggest a biological coding strategy in which the visual system represents actions and events by assembling a set of basic elements in a hierarchical way. As an example for this strategy, our recent recording in the mouse's primary visual cortex suggests that patterns of sequential firing of a population of V1 neurons can encode various movements of a set of basic stimulus features (Figure S2).

By comparison, global representations of actions and events [11], [12] by the computational modeling have not made use of hierarchical neuronal encoding properties observed in the brain, but rather employed the spatial-temporal volumes of actions and events as a whole. On the other hand, current modeling on local representations of actions and events [13]–[19], including cuboids descriptors, histograms of optical flow, are not compatible with the neuronal response properties. For instance, the basic spatial-temporal features in these local representations are not similar to the features to which V1 neurons are tuned. Moreover, the basic features used in these local representations are discriminative features but are not intended to encode natural actions in a hierarchical way.

In the NASs obtained here, there are two layers. In the first layer, the basic spatial-temporal features are ICs, similar to the features to which V1 neurons are tuned. The feature element *a_i_* of each patch sequence is the root mean square of the amplitudes of the ICs in an IC cluster (Equation (2)). In the second layer, each structural cluster in the space of feature vector contains a combination of the IC clusters, some of which may be more dominant than others. Thus, each NAS is characterized by a set of IC clusters at multiple spatial-temporal scales. At this time, we can only speculate the possible neural mechanisms for this coding scheme of natural actions. First, the feature element *a_i_* corresponding to an IC cluster cannot be encoded by a single V1 neuron but may be expressed as a nonlinear function of the responses of a set of V1 neurons whose tuning to orientation, speed, direction, frequency, and phase are similar (since the ICs in an IC cluster have similar parameters). This neural model of *a_i_* would be a straightforward generalization of the LN model to the population level. Second, an NAS, which usually contains multiple IC clusters, may not be encoded by a single MT neuron but may be expressed as a nonlinear function of the responses of a set of MT neurons whose tuning characteristics are broadly similar (since the dominant IC clusters in a structural cluster are broadly similar). This neural model of NAS can be viewed as a critical generalization of the MT neuron model [48] to the population level. Thus, it is conceivable that the population coding strategy can give rise to various NASs along the visual pathways.

### Future directions

Our present study can be extended in three major ways. The first extension is to develop parameterized, probabilistic models of NASs. Because of the limited sizes of the datasets used in this study, we did not attempt to develop probabilistic models on the NASs. For this purpose, we are currently developing a large dataset of human actions. With parameterized, probabilistic models of NASs, we will be able to perform a range of statistical analyses on NASs and develop a hierarchical, probabilistic model of action recognition. The second extension is to develop combinatorial principles for encoding natural human actions. These principles, acting like a stochastic grammar, similar to image grammars [49], specify the spatial and temporal concatenations of NASs for action encoding, and NASs are the probabilistic code words in this action grammar. The third extension is to combine this model with our models of motion saliency [50] and object detection in natural contexts [24] to categorize scenes and detect objects, animals and humans, and their actions simultaneously. These directions need to be pursued in the future.

## Materials and Methods

### Datasets of human actions

We used two video datasets of human actions, the KTH dataset [14] and the Weizmann dataset [11]. The KTH dataset contains 2,391 videos of six actions, boxing, handclapping, handwaving, jogging, running, and walking, performed by 25 human subjects. Each video contains repeated executions of a single action and has a resolution of 160×120 pixels and a frame rate of 25 frames/second. These videos were recorded under four conditions: outdoor, outdoor with variations in scale, outdoor with different cloths, and indoor. Figure 2A shows 4 samples of each action in the dataset.

The Weizmann dataset contains 90 videos of 10 actions performed outdoors by 9 human subjects, including bend (bending), jack (jumping jack), jump (jumping forward on two legs), pjump (jumping in place on two legs), run (running), side (galloping sideways), skip (skipping), walk (walking), wave1 (waving with one hand), and wave2 (waving with two hands). Each video contains repeated executions of a single action and has a resolution of 180×144 pixels and a frame rate of 25 frames/second. Figure 2B shows one sample of each action in this dataset.

### Training and cross-validation

To test our models of action recognition on the KTH dataset, we used the separation of the training set (subjects: 1, 4, and 11–25) and testing set (subjects: 2, 3, 5–10, and 22) coming with the dataset. Cross-Validation (CV) is a way to keep in control over-fitting and is widely used in pattern recognition. We used LOOCV on the training set to select model parameters, which included: 1) the number of clusters obtained by the K-means method, 2) the thresholds, Mc and Nc, for selecting NASs, 3) the partition grids for the SVM classifier, and 4) the parameters of the LDA and SVM classifiers. In this procedure, we tested on the video samples of a single subject using the remaining samples as the training set. We repeated this procedure until the video samples of all subjects were tested. To test our models on the Weizmann dataset, we used LOOCV on the whole dataset since this dataset is small.

### Pre-processing

We used Dollar's cuboids detector and descriptor on both KTH and Weizmann dataset as a baseline method. We set the spatial scale to 2 and the temporal scale to 3 in the cuboids detector and the size of cuboids to 13 (space)×13 (space)×19 (time). To detect points of interests in space-time volumes for compiling NASs, we used the cuboids detector, defined as the local maxima of the response function. At the selected points of interests, we sampled sequences of circular patches of sizes of 13×13×11, 25×25×21, and 49×49×31 and aligned to each other the centers of the middle frames in the patch sequences (i.e., 6^th^, 11^th^, and 16^th^).

### Testing on KTH dataset

We randomly sampled 6×10^5^ patch sequences, performed ICA on the sequences, and obtained 1,200 ICs that accounted for 99.5% of the variance of the patch sequences. We fitted Gabor functions to the 1,200 ICs, converted the fitted parameters into pre-set intervals (i.e., the scale of the Gaussian envelope to [0, +∞), the orientation of sinusoid carrier to [0, π), and the phase of the sinusoid carrier to [0, 2π)). However, we did not convert the location of the Gaussian envelope. We then collapsed the ICs into 480 IC clusters using the K-means method with the Euclidean distance as a function of the parameters of the Gabor function.

To compile NASs, we randomly selected 5×10^5^ patch sequences, mapped them to the 480 IC clusters, and collapsed them to 1,000 structural clusters at each scale using the K-means method. The distance used in this step was the Euclidean distance between the root mean square of the amplitudes of the ICs in the IC clusters. We call all the patch sequences that share the same structural clusters at the three spatial-temporal scales a NAS. The total number of NASs is 193,600.

To select NASs for the SVM classifier, we set the thresholds, Mc and Nc, to 2 and 400 respectively. The total number of selected NASs is 4,998. We used the 1- χ^2^ kernel in the SVM classifier and set C to 0.125. Also, we partitioned the video sequences into 3-1-1 grids (i.e., 3 equal grids in the vertical dimension and 1 grid in the horizontal and temporal dimensions). For LDA classifier, we set the thresholds Mc and Nc to 3 and 300 respectively. The total number of the selected NASs is 4,349 and the number of topics is 50.

### Testing on Weizmann dataset

We randomly sampled 1.2×10^6^ patch sequences, performed ICA on the sequences, and obtained 1,200 ICs that accounted for 99.9% of the variance of the patch sequences. We fitted Gabor functions to the 1,200 ICs, converted the fitted parameters (see above), and collapsed the 1,200 ICs into 440 clusters using the K-means method with the Euclidean distance. To compile NASs, we randomly selected 23,000 samples, mapped them to the 440 IC clusters, and collapsed them to 700 structural clusters using the K-means method (see above). The total number of NASs is 11,540.

To select NASs for the SVM classifier, we set the thresholds, Mc and Nc, to 1 and 500 respectively. The total number of selected NASs is 6,392. We used the 1- χ^2^ kernel in the SVM classifier and set C to 0.125. For this dataset, we used grid 1-1-1. For LDA classifier, we set the thresholds, Mc and Nc, to 0 and 100 respectively. The total number of the selected NASs was 1,526 and the number of topics is 30.

## Supporting Information

Figure S1 **Representative visual receptive fields recorded from the mouse primary visual cortex.** The visual receptive fields were recorded by using 64-channel recording array in stereotrode format. The mouse's head was fixed to a crossbar, and standing or running on tread mill.
(TIF)

Figure S2 **Neural correlates of action recognition responses in the mouse's V1 cortex.** (A–C), Neurons #1, 2, and 3 have the preferred orientation at 45°, 67.5°, and 135°, respectively, based on their responses to drifting grating stimuli. Eight orientations were given as shown. (D), Sequential firings of Neurons #1, 2, and 3 may provide a mechanism for computing upper body and head motion information along the visual pathway during the action recognition of “standing up”.
(TIF)
