# Supplementary figures and images for: Robust Action Recognition Using Multi-Scale Spatial-Temporal Concatenations of Local Features as Natural Action Structures
Source: PLoS One. 2012 Oct 4;7(10):e46686. doi: 10.1371/journal.pone.0046686 (PMC3464264; doi:10.1371/journal.pone.0046686)

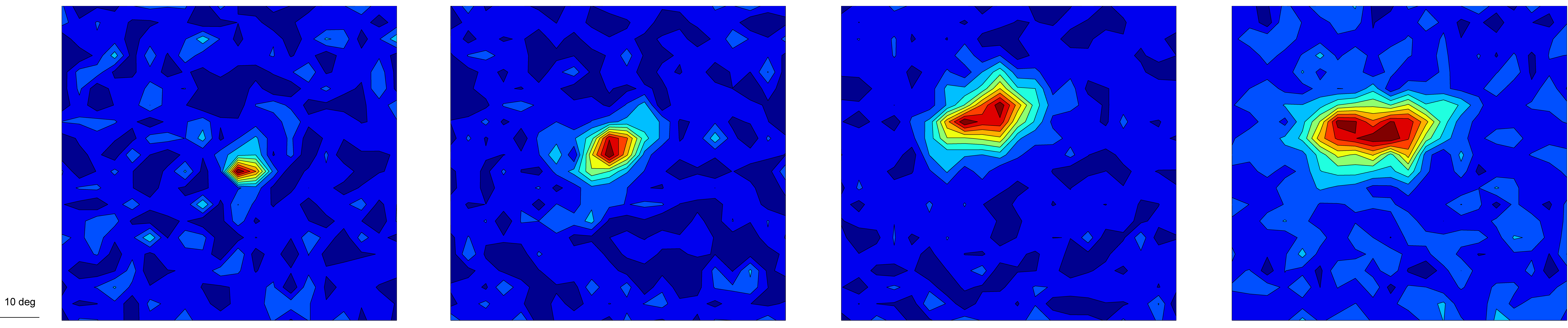

Supplement: Figure S1 — Representative visual receptive fields recorded from the mouse primary visual cortex. The visual receptive fields were recorded by using 64-channel recording array in stereotrode format. The mouse's head was fixed to a crossbar, and standing or running on tread mill. (TIF) [file pone.0046686.s001.tif]

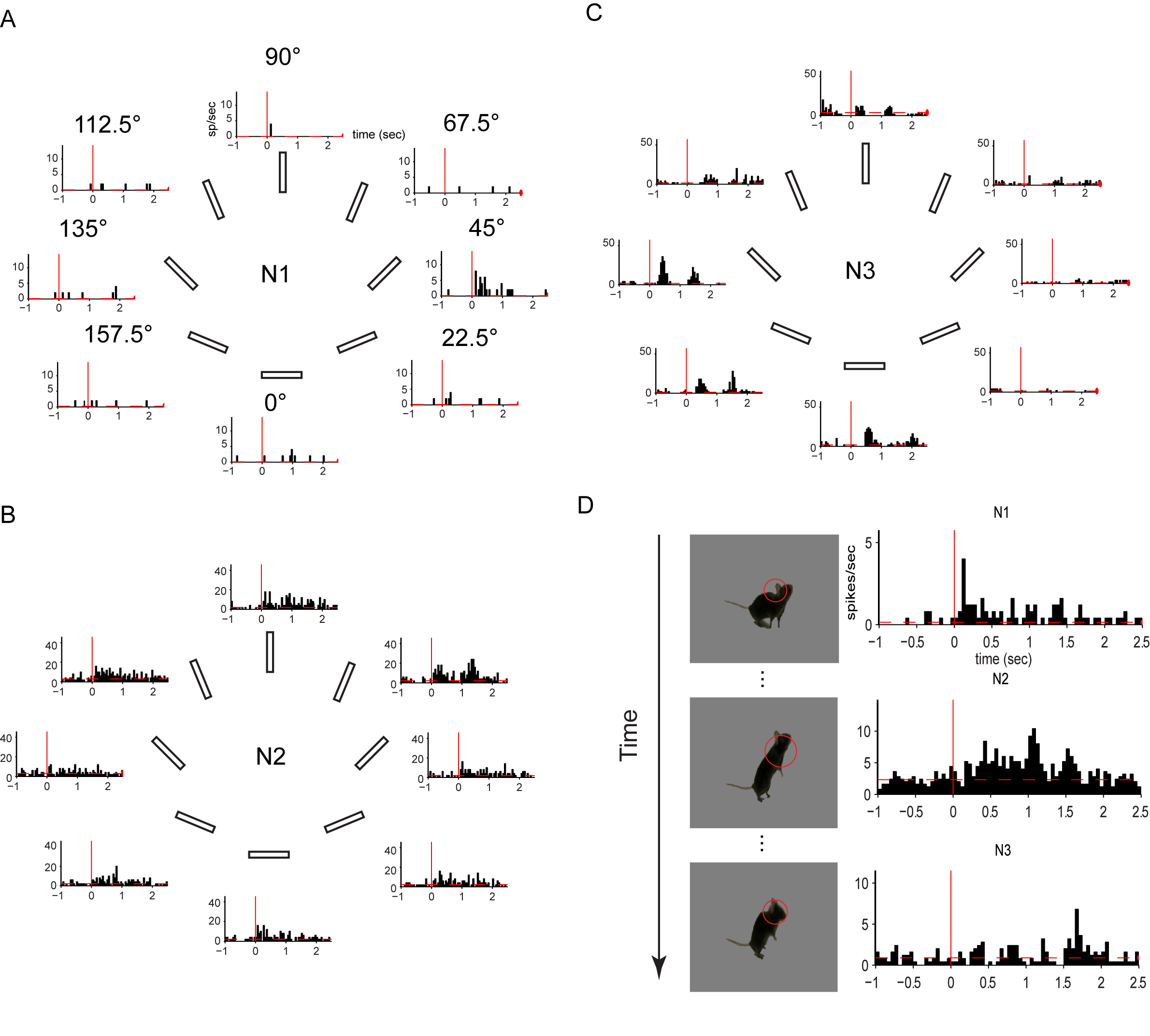

Supplement: Figure S2 — Neural correlates of action recognition responses in the mouse's V1 cortex. (A–C), Neurons #1, 2, and 3 have the preferred orientation at 45°, 67.5°, and 135°, respectively, based on their responses to drifting grating stimuli. Eight orientations were given as shown. (D), Sequential firings of Neurons #1, 2, and 3 may provide a mechanism for computing upper body and head motion information along the visual pathway during the action recognition of “standing up”. (TIF) [file pone.0046686.s002.tif]
